# Using targeted vouchers and health equity funds to improve access to skilled birth attendants for poor women: a case study in three rural health districts in Cambodia

**DOI:** 10.1186/1471-2393-10-1

**Published:** 2010-01-07

**Authors:** Por Ir, Dirk Horemans, Narin Souk, Wim Van Damme

**Affiliations:** 1 Provincial Health Department, Ministry of Health, Siem Reap, Cambodia; 2 Department of Public Health, Institute of Tropical Medicine, Antwerp, Belgium; 3 Provision of Basic Health Services, Belgian Technical Cooperation, Phnom Penh, Cambodia; 4 Provision of Basic Health Services Kampong Cham, Belgian Technical Cooperation, Kampong Cham, Cambodia

## Abstract

**Background:**

In many developing countries, the maternal mortality ratio remains high with huge poor-rich inequalities. Programmes aimed at improving maternal health and preventing maternal mortality often fail to reach poor women. Vouchers in health and Health Equity Funds (HEFs) constitute a financial mechanism to improve access to priority health services for the poor. We assess their effectiveness in improving access to skilled birth attendants for poor women in three rural health districts in Cambodia and draw lessons for further improvement and scaling-up.

**Methods:**

Data on utilisation of voucher and HEF schemes and on deliveries in public health facilities between 2006 and 2008 were extracted from the available database, reports and the routine health information system. Qualitative data were collected through focus group discussions and key informant interviews. We examined the trend of facility deliveries between 2006 and 2008 in the three health districts and compared this with the situation in other rural districts without voucher and HEF schemes. An operational analysis of the voucher scheme was carried out to assess its effectiveness at different stages of operation.

**Results:**

Facility deliveries increased sharply from 16.3% of the expected number of births in 2006 to 44.9% in 2008 after the introduction of voucher and HEF schemes, not only for voucher and HEF beneficiaries, but also for self-paid deliveries. The increase was much more substantial than in comparable districts lacking voucher and HEF schemes. In 2008, voucher and HEF beneficiaries accounted for 40.6% of the expected number of births among the poor. We also outline several limitations of the voucher scheme.

**Conclusions:**

Vouchers plus HEFs, if carefully designed and implemented, have a strong potential for reducing financial barriers and hence improving access to skilled birth attendants for poor women. To achieve their full potential, vouchers and HEFs require other interventions to ensure the supply of sufficient quality maternity services and to address other non-financial barriers to demand. If these conditions are met, voucher and HEF schemes can be further scaled up under close monitoring and evaluation.

## Background

The Millennium Development Goal 5 (MDG5) aims to reduce the maternal mortality ratio by three quarters between 1990 and 2015 [1]. However, progress towards this goal has been disappointing. The maternal mortality in many countries, especially in Sub-Saharan Africa and Asia, remains high with huge poor-rich inequalities [2,3]. Targeting effective maternal health interventions to the most vulnerable, especially the rural poor populations, is considered essential to achieve MDG5 [3]. However, there are few examples of successful maternal health interventions aimed at the poor [4].

Effective strategies to reduce maternal mortality are well known nowadays. Ensuring access to skilled birth attendants and emergency obstetric care are two priority interventions fundamental to the prevention of avoidable maternal deaths [5,6]. Some scholars propose that the main priority for developing countries should be to ensure that women deliver in health centres [7,8]. It has been estimated that the presence of skilled attendants at delivery could reduce maternal deaths by 13 to 33 percent [9]. However, existing programmes aimed at improving maternal health in many countries have been found to be ineffective in preventing maternal mortality and often fail to reach the poor [10]. Besides transport and time costs, formal and informal fees in public health services constitute a substantial financial barrier for poor women to access maternal health services [11].

The Cambodian maternal mortality ratio is among the highest in South and South-East Asia, at 472 maternal deaths per 100,000 live births [12]. Despite considerable progress, maternal health service indicators remain low with a large poor-rich gap. The Cambodia Demographic and Health Survey 2005 [13] showed that 43.8% of deliveries were assisted by trained health professionals. Nevertheless, only 21.5% occurred in a health facility. More than half of women still delivered with a traditional birth attendant at home. Furthermore, only 20.7% of deliveries among women in the lowest socio-economic quintile were attended by trained health professionals and 6.5% in a health facility. The figures for the richest quintile were 89.9% and 67.4% respectively. Poor pregnant women have to overcome many barriers to deliver in a health facility with trained health professionals [14].

To tackle financial barriers to access, developing countries are implementing various demand-side approaches to financing health care that subsidize the consumers directly. Among these, vouchers are considered a potentially effective means of targeting health services to specific population groups [15-18]. Vouchers for health are defined as "a financing mechanism for subsidizing the price of health services and products to target population groups, with the goal of improving access to and utilization of those services and products" [19]. So far, documented evidence on the success of vouchers has been limited, especially in the field of maternal care [20-24]. In Cambodia, the Ministry of Health and the Belgian Technical Cooperation initiated a voucher scheme in 2007 to complement an existing Health Equity Fund (HEF) scheme for improving access to safe delivery for poor women in three rural health districts, alongside other strategies such as performance-based contracting and delivery incentive schemes. Like vouchers, HEFs constitute a demand-side financing mechanism that promotes access to priority public health services for the poor. Available evidence suggests that HEF can effectively improve access to health services for the poor and protect them from the burden of health care costs [25-27].

The aim of this paper is to assess to what extent voucher and HEF schemes have improved access to skilled birth attendants for poor women. This study was conducted in three rural health districts in Cambodia, as part of an international research project, "Poverty and Illness" http://www.povill.com. We examined the trend of deliveries in public health facilities as percentage of the expected number of births. An operational analysis of the voucher scheme was carried out to assess its effectiveness at different stages of operation. By assessing the effectiveness of the voucher and HEF schemes in improving access to skilled birth attendants for poor women we can draw lessons for their further improvement and scaling-up in order to reduce maternal mortality in Cambodia.

## Methods

### Study setting and interventions

Kampong Cham is the biggest province in Cambodia with a total population of 1,680,000. The public health system in this province consists of ten operational health districts (ODs), each with a referral hospital and several health centres. The provincial hospital is the referral hospital of Kampong Cham OD, located in the provincial town. All the public health facilities receive free drugs and medical supplies, staff salaries, and a budget for running costs (which make up about 60-70% of the total recurrent costs) from the government. In addition, they charge fees from patients. Along with the public health system, there are numerous and often unregulated private practices. According to the Cambodia Demographic and Health Survey 2005, 12.3% of births in Kampong Cham province took place in a health facility and only 8.2% occurred in public facilities. About 53% of women delivered at home with traditional birth attendants [13].

The study was conducted in three of the ten ODs in Kampong Cham province, namely Cheung Prey, Chamkar Leu and Prey Chhor. There are three referral hospitals (without operation theatres for surgical interventions) and 42 health centres, serving a total population of approximately 538,000. Surgical cases, including caesarean sections, are referred to the provincial hospital. Since 2005, the Ministry of Health and the Belgian Technical Cooperation have implemented several health financing schemes, including Health Equity Fund (HEF), vouchers, and performance-based contracting (PBC) to improve access to basic health services for the population, especially the poor, in the three ODs. At the end of 2007, the government introduced a delivery incentive scheme nation-wide to boost deliveries in public health facilities. Through this scheme, midwives and other health personnel receive a government incentive of USD12.5 for each live birth attended in a referral hospital and USD15 in a health centre on top of the fees they charge from patients.

The HEF scheme (See Additional file 1) started in late 2005 in the three district hospitals in the study area to improve access for the poor to hospital care services. The management of the HEF scheme was entrusted to two non-governmental organisations (NGOs), acting as a third party purchaser. NGO staff interview potentially poor patients at the hospitals to determine their eligibility for HEF assistance, using a predefined questionnaire and eligibility criteria (See Additional file 1). On the basis of the total index scores, the interviewees are then classified into three categories of eligibility: very poor, poor and non-poor. The latter category is excluded from HEF assistance. According to the eligibility category, patients receive a full or partial benefit package, including payment for hospital user fees, payment for the cost of transportation to the health centre or hospital, food allowance during the hospitalization, and funeral cost in the event of death.

The voucher scheme was launched in Cheung Prey, Prey Chhor and Chamkar Leu operational health districts (ODs) in February, June and July 2007, respectively. The objective was to improve access to safe delivery for poor women, thereby contributing to the reduction of maternal and newborn mortality and morbidity. The management of the voucher scheme was sub-contracted to the NGOs operating HEF in the area, as voucher management agencies (VMA). The voucher recipients are poor pregnant women in the catchment area. Public health centres are selected to provide health services to the voucher recipients. To ensure sufficient quality for safe delivery, only 30 of the 42 health centres in the three ODs were selected on the basis of three criteria: they (1) can provide the minimum package of services (as recommended by the Ministry of Health for a health centre), (2) have at least one skilled midwife available in time of need, and (3) have a record of relatively high utilisation for antenatal care and delivery. Only these health centres were thus contracted to provide maternal services to voucher recipients in a timely and professional way.

Poor pregnant women are identified by local health volunteers and VMA staff at home, using the same pre-defined questionnaire and eligibility criteria as for HEF. Each eligible poor woman receives a voucher with five detachable coupons, which entitle her to free services at the health centre (for three antenatal care visits, delivery and one postnatal care visit) and transportation costs for five round trips between her home and the health centre, and for referrals from the health centre to the referral hospital in case of complications. User fees and other related costs at referral hospitals are paid for by the HEF. Voucher recipients are encouraged to use all five coupons for their pregnancy, but they are free to use only one or a few of them. The vouchers are only valid for the current pregnancy. At the end of each month, the VMA pay the contracted health centres on the basis of the number of coupons and the price of user fees (about USD7.5 for a normal delivery and USD0.25 for each antenatal and postnatal care visit). The VMA provide cash advances to the contracted health centres to pay for the transportation cost of voucher recipients using a pre-defined price-list. The list estimates the transportation cost for each village in the catchment area to the health centre, taking USD0.1 per kilometre, the estimated rate for moto-taxis, as the unit-based fare. For monitoring purposes, the VMA collected routine data on the number of poor pregnant women identified, the number of vouchers distributed, the utilisation of vouchers for ANC, delivery, postnatal care and referral services, the costs of services provided through the voucher schemes and the number of deliveries supported by HEF at hospitals.

The performance-based contracting (PBC) scheme started in late 2005 and was gradually expanded to all government health facilities and management bodies in the three ODs as a strategy to address the vicious cycle of underpaid health staff, and poorly performing and thus under-utilised health services. This strategy was inspired by the 'Cambodian New Deal' experiment in Sotnikum, which is described in detail elsewhere [28,29]. In the PBC arrangements, contracted facilities receive financial incentives related to certain process and output indicators. In addition, they also receive support for staff capacity building, quality improvement and basic drug and medical supplies. As a result, the performance of the contracted facilities has improved considerably and a minimum quality (24 hour services and absence of informal fees) is now more or less ensured.

In two other ODs in Kampong Cham province, Memot and Ponheakrek, the Ministry of Health and its development partners implemented a special "contracting" scheme initially in Memot in 1999 as a first phase, and then in Ponheakrek in 2004 as a second phase. Several studies have described the first phase of this contracting model and demonstrated its effectiveness [30,31]. In general, it is similar to the PBC in the three study ODs. The only difference is that the management of the ODs is completely outsourced to an international organization as contractor and that the performance-based part of staff income is much higher than in the PBC model. In four other rural ODs, there were no major interventions during the study period, apart from the delivery incentive scheme.

### Conceptual framework

**Figure 1:**
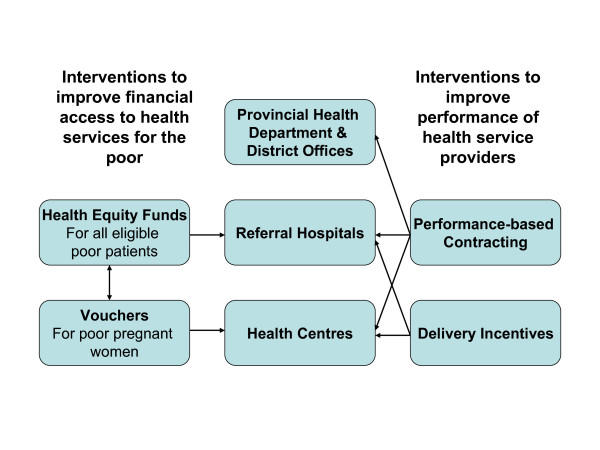
**Strategy used to improve access to skilled birth attendants**.

Poor people may encounter numerous barriers to accessing health care [32,33]. User fees are one of the main barriers to accessing government health services in low-income countries [34]. In Cambodia, previous studies have identified several access barriers related to distance, costs, quality of care, knowledge of users, and socio-cultural practices [25,26,35]. In addition to costs, low staff income (which often induces attitude problems or even absence of the midwife at the health facility) has been found to be one of the main causes of the low percentage of deliveries in public health facilities [14]. Figure 1 shows how different interventions in the three study ODs address the above barriers.

The hospital-based HEF that identifies poor patients and pays user fees and other access-related costs on their behalf improves financial access for the poor to hospital care, including delivery [25-27]. Although in general health centre user fees are not a big barrier to access in Cambodia [36], the fee for delivery could nevertheless constitute a major financial barrier for the poor, especially when combined with transport cost [14]. The voucher scheme, which is designed as a health centre-based HEF for maternal services, improves financial access for poor women to maternal care, including delivery, at the health centre.

The PBC and the delivery incentive scheme address the issue of low staff income through performance and output-based cash incentives. The PBC helps improve the general performance of providers whereas the delivery incentive scheme focuses on the midwife and delivery. More generally, these interventions can also improve quality of care and partly address financial barriers by preventing informal payments [31,37].

### Data collection

In this case study, we used a combination of methods to collect quantitative and qualitative data. We collected data on the number of vouchers distributed and the number of voucher and HEF beneficiaries between 2006 and 2008 from the database and reports of the VMA and HEF agents.

Data on deliveries in public health facilities (facility deliveries) in the three study ODs were extracted from the routine health information system. For comparison, similar data were also gathered for other ODs in Kampong Cham province. In the routine health information system, data on deliveries in the facilities are collected every month by the health facilities in consultation with community representatives who gather information on deliveries during the previous month in all villages in the catchment area of the health centre.

To calculate denominators for the assessed indicator, facility deliveries as percentage of the expected number of births, we used the population figure estimated by the recent census and the crude birth rate of 25.9‰ for rural areas as estimated by the Cambodia Demographic and Health Survey 2005 [13]. This crude birth rate seems to hold true for 2006, 2007 and 2008, as the trend in contraceptive prevalence rate indicated by the health information system remained relatively stable throughout this period. If this trend had changed, the magnitude of the change would not have been very large. However, the fertility rate among poor women was estimated to be higher than among the general population. Therefore, we estimated the crude birth rate among poor women, the target group of voucher and HEF schemes, at 27‰. The recent identification of poor households in the study area found 26% of the households to be poor and eligible for HEF and vouchers.

Qualitative data were collected through focus group discussions and key informant interviews. We conducted nine focus group discussions in late 2007 with a total of 87 voucher recipients. Five groups included 51 voucher recipients who did not use their vouchers for delivery (non-user group) whereas the four other groups included 36 voucher recipients who used their vouchers (user group). Participants were randomly selected from the list of voucher recipients and beneficiaries at health centres, the non-user group from health centres with low utilisation rates of vouchers for delivery and the user group from health centres with high utilisation rates of vouchers. The aim of the focus group discussions was to understand reasons for use and non-use of vouchers. For similar purposes, the first author conducted in-depth interviews with 20 voucher recipients, both voucher users and non-users, in early 2009. He also interviewed 18 key informants, including village health volunteers, traditional birth attendants, health centre midwives, health centre chiefs, district and provincial chiefs of maternal and child health programmes, managers of the two VMA and HEF implementing NGOs and key staff of the Belgian Technical Cooperation to gain insight in the implementation process and the effectiveness of voucher and HEF schemes.

### Data analysis

WHO defines a skilled attendant as "an accredited health professional - such as a midwife, doctor or nurse - who has been educated and trained to proficiency in the skills needed to manage normal (uncomplicated) pregnancies, childbirth and the immediate postnatal period, and in the identification, management and referral of complications in women and newborns" [38]. In the study area, deliveries in public health facilities (facility deliveries) were attended by trained midwives and other health personnel, who are considered skilled birth attendants.

In order to assess the effectiveness of vouchers and HEFs in improving access to skilled birth attendants, an indicator on deliveries in public health facilities as percentage of the expected number of births was computed in MS excel. This indicator was calculated for the nine rural ODs in Kampong Cham province operating under the management umbrella of the Provincial Health Department. We first assessed the trend of this indicator between 2006 and 2008 in the three study ODs and then compared it to the situation in two other groups of ODs without voucher and HEF schemes: respectively, a group of two ODs with special contracting and the delivery incentive scheme, and a group of four ODs with only the delivery incentive scheme. To avoid bias, we excluded Kampong Cham OD from the comparison, as this OD covers the provincial town and the urban area, and the provincial hospital providing referral services for the whole province is also based there.

The operational analysis of the voucher scheme focused on three main stages: health centre selection, voucher distribution and voucher utilisation. The last-named was analysed among voucher recipients in 2007, as some of the voucher recipients in 2008 had not yet delivered at the time of the study.

The qualitative data from the focus group discussions and in-depth interviews were manually coded, grouped and analysed.

### Ethical consideration

This study is part of the EC-funded Poverty and Illness research project, which received ethical approval from the Cambodian National Ethics Committee for Health Research on 10 August 2007 with reference number 063 NECHR. Verbal consent was obtained from each participant and respondent prior to the focus group discussion and interview.

## Results

### Utilization of vouchers and HEF

A total of 2,725 vouchers were distributed in the three health districts within less than two years of operation. During this period, 2,062 vouchers were used by poor pregnant women for ANC1, 1,498 for ANC2, 1,140 for ANC3, 1,280 for delivery and 684 for postnatal care. Of the 1,280 voucher users for delivery, 215 delivered in referral hospitals; 63 of them were referred by health centres whereas 152 others went straight to the hospitals possibly after advice given by the health centres during ANC visits.

**Figure 2:**
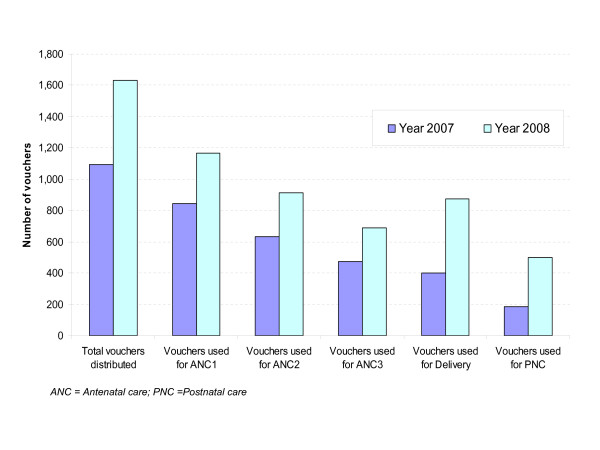
**Vouchers distributed and used in 2007 and 2008**.

Figure 2 presents the total number of vouchers distributed and used in 2007 and 2008. The comparison of the figures shows a significant increase in the number of vouchers distributed and used for all recommended services, especially delivery. However, in both years the difference between the number of distributed and used vouchers remains large, indicating that many distributed vouchers were not used.

In 2006, HEF supported 132 poor pregnant women who delivered at the three district hospitals. The respective figures for 2007 and 2008 were 346 and 549; these included the voucher holders.

### Facility deliveries in the three study ODs

Among the total of 5,611 facility deliveries in the three study ODs in 2008, 4,391 (78.3%) happened in health centres and 1,220 (21.7%) in referral hospitals. Vouchers supported 876 (19.9%) of the total health centre deliveries while HEF supported 549 (45%) of the total hospital deliveries. In total, 1,425 poor pregnant women benefited from the voucher and HEF schemes, which accounts for 25.4% of the total number of facility deliveries. Vouchers and HEFs financed 11.4% of the expected number of births in the three study ODs (estimated at 12,485) and 40.6% of the expected number of births among poor women (3,509) in 2008.

**Figure 3:**
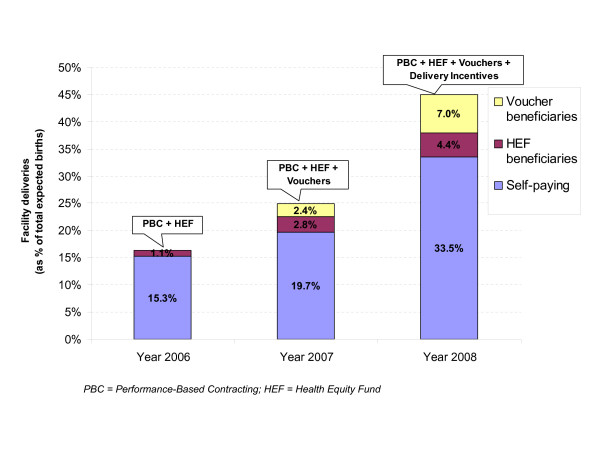
**Facility deliveries by type of beneficiary in the three ODs between 2006 and 2008**.

Figure 3 shows that facility deliveries as percentage of the expected number of births in the three ODs increased sharply from 16.3% in 2006 to 24.9% in 2007 and 44.9% in 2008, and this increase was not only for voucher and HEF beneficiaries, but also for self-paid deliveries. Facility deliveries of voucher beneficiaries increased by 195.9% within two years, from 2.4% in 2007 to 7% in 2008, while the figures for HEF beneficiaries and self-paid deliveries increased by 58.1% and 69.8% respectively within the same period. The highest increase in facility deliveries was observed in 2008 when all three interventions were put in place.

### Comparison of facility delivery trends in the three study ODs and two other groups of ODs

**Figure 4:**
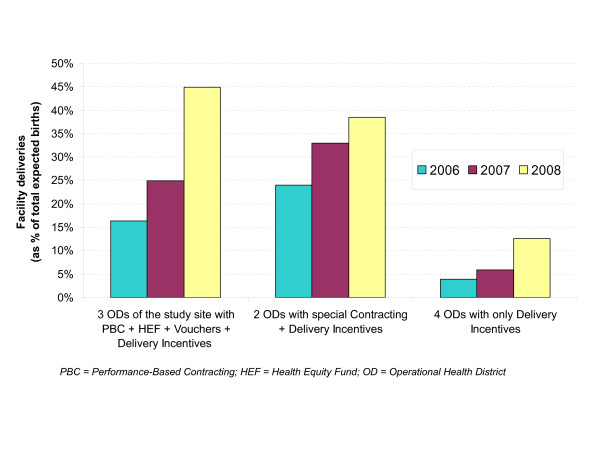
**Comparison of facility deliveries in three groups of ODs in Kampong Cham between 2006 and 2008**.

Figure 4 compares the facility deliveries in the three study ODs to those in two other groups of ODs between 2006 and 2008. The figure shows that the facility deliveries increased in the three groups of ODs over this period. The absolute increases between 2006 and 2008 were 28.6%, 14.5% and 8.6% of the expected number of deliveries, respectively, for the group of study ODs, the group of two ODs with special contracting, and the group of four ODs with the delivery incentive scheme only. In the last group, the percentage of facility deliveries also increased substantially in 2008, but the increase was less pronounced than in the other two groups.

### Operational analysis of the voucher scheme

The operational process of the voucher scheme can be divided into three stages: (1) health centre selection, (2) voucher distribution and (3) voucher utilisation.

**(1) Health centre selection**. By 2008, only 30 (71.4%) of the 42 health centres in the three ODs had been selected and included in the voucher scheme. Twelve health centres and their catchment villages were not covered because they did not meet the selection criteria (six health centres lacked proper infrastructures). Pregnant women living in the catchment areas of these twelve health centres were thus automatically excluded from the voucher scheme.

**(2) Voucher distribution**. Voucher distribution started with the pre-selection of potentially poor pregnant women in the target villages by village health volunteers. These women were later interviewed by VMA staff. On the basis of the schedule set for 2007, the VMA staff was supposed to make a total of 894 visits to the 329 villages in the catchment areas of the 30 contracted health centres. In reality, only 545 (60.9%) of the scheduled visits took place.

**(3) Voucher utilisation**. Analysis of the 1,093 poor pregnant women who received vouchers in 2007 shows that 855 (78.2%) of them had used their vouchers for ANC1, 665 (60.8%) for ANC2, 501 (45.8%) for ANC3 and 487 (44.6%) for delivery. Therefore, more than half of the voucher recipients did not make use of their voucher for delivery.

### Results from focus group discussions

None of the 87 women participating in the focus group discussions had delivered in the health centre prior to the introduction of the voucher scheme, although about half of them had previously sought ANC at the health centre. Almost all the women had used vouchers to seek ANC at the contracted health centres at least once, even those who had not used their voucher for delivery (the non-user group).

All the women participating in the user group were in general satisfied with the services provided at the health centres. They reported three main reasons for using their vouchers for delivery at health centres. First, with a voucher they could get free care and some money to pay for transportation costs. Second, they felt safer when delivering at the health centre (compared to home deliveries with traditional birth attendants). Third, they could immediately get their child vaccinated after the delivery at the health centre.

Women participating in the non-user group reported several reasons for the non-use of their vouchers for delivery at health centres. Transportation and intra-household constraints were mentioned as the two main reasons. First, some women lived in remote areas far away from the health centres. Although they knew that transportation costs would be paid for by the voucher scheme, they could seldom find appropriate means of transport when the deliveries happened in the middle of the night. If they did manage to find transport, they anticipated that the price would be much higher than the day time price approved by the voucher scheme. They therefore feared that such higher costs would not be fully covered by the voucher scheme. Second, several intra-household constraints made it difficult for some poor pregnant women to leave their home. Many women claimed that if they came to deliver at health centres, nobody would look after their house and take care of their children or that nobody could accompany them to health centres. In addition, many of them expressed dissatisfaction with health centre services and staff. Some women reported poor staff attitudes and extra payments hinted by midwives. Some doubted the midwife's availability at night for delivery.

### Results from key informant interviews

All the key informants observed a significant improvement in facility deliveries in the study area. Many of the village health volunteers and traditional birth attendants interviewed claimed that there were almost no home deliveries any more in their villages. The traditional birth attendants referred all pregnant women to health centres for delivery. Other key informants also confirmed this. They reported several reasons for this improvement. First, poor pregnant women who received a voucher could now go to the health centre without having to overcome financial barriers. Second, thanks to the cash incentives from the PBC and delivery incentive scheme, midwives and health centre personnel had become more committed to ensuring 24-hour services at health centres and to providing more health education to promote facility deliveries during outreach activities. Third, village health volunteers and traditional birth attendants also received cash incentives from the health centre for referrals of pregnant women for delivery at the health centre. Fourth, the district and provincial health management teams applied stronger monitoring and stricter rules for 24-hour services. Informal payments were no longer allowed.

## Discussion

The results show that the number of facility deliveries increased sharply after the introduction of the voucher and HEF schemes. This increase was much greater in the three study ODs than in other ODs without voucher and HEF schemes. In 2008, voucher and HEF beneficiaries accounted for 40.6% of the expected number of births among the poor. Furthermore, many of the voucher and HEF beneficiaries delivered in public health facilities for the first time, as indicated by the focus group discussions. Poor pregnant women who made use of vouchers to deliver at health centres and hospitals had previously delivered at home with traditional birth attendants. This suggests that in the three study ODs in Kampong Cham, voucher and HEF schemes indeed improved access to facility deliveries for poor women.

However, we should be cautious when interpreting these data, especially about the impact of the voucher and HEF schemes on improved access to skilled birth attendants for poor women. First, the increase in facility deliveries could represent just a shift from deliveries attended at home by trained health professionals. Second, several health financing schemes under implementation in the study area and other socio-economic factors might have contributed to the increase in facility deliveries. Third, it is rather early to perceive the full effect of the voucher scheme since it has only been fully implemented for less than two years.

An increase in facility deliveries could represent just a shift from deliveries attended at home by trained health professionals (skilled attendants) to facility deliveries. Hence, analysis of the trend in the percentage of deliveries at home attended by trained health professionals is necessary. Unfortunately, we do not have reliable data for that analysis. According to the Cambodia Demographic and Health Survey 2005 [13], almost all women in rural Cambodia (83%) delivered at home and most (60%) of them did so with traditional birth attendants. The respective figures were even higher for the poorest quintile (93% and 78% respectively). Key informants estimated that these findings remain valid for the current situation in the study area. They said that it is usually expensive to have a delivery attended by a trained health professional at home. Poor women cannot afford such an expensive fee. It is cheap and easy for them to deliver at home with traditional birth attendants. Therefore, almost all home deliveries among poor women are attended by traditional birth attendants. This suggests that voucher and HEF schemes increase deliveries assisted by skilled birth attendants by enabling poor women who used to deliver at home with traditional birth attendants to deliver in public health facilities.

In the study area, besides vouchers and HEFs, several other factors could have contributed to the increase in facility deliveries. The overall increase, especially the increase in self-paid facility deliveries, can be explained by the overall improvement in provider performance. Thanks to the PBC and the delivery incentive scheme, midwives were more regularly present at the health facility and changed their behaviour for the better. One could claim that the sharp increase in facility deliveries (which included voucher and HEF beneficiaries) in the three study ODs in 2008 was due to the introduction of the delivery incentive scheme; the incentive of USD12.5-USD15 per attended delivery provided through this scheme is about twice the price of the fee for delivery paid for by voucher, HEF and patients. However, comparison among the three groups of ODs shows that the delivery incentive scheme does not suffice if implemented alone. The comparison also shows that the combination of special contracting and the delivery incentive scheme can achieve relatively good results. However, results attained with the voucher and HEF schemes tend to be better. Furthermore, the PBC and the delivery incentive scheme do not address financial barriers of access for pregnant women, especially poor pregnant women. Even if the health centre and hospital offer good quality delivery services, poor pregnant women who cannot afford to pay the cost of transport and user fees will not go there. In a Cambodian survey, other factors such as improved road access and increasing awareness among women of how to deliver safely were also found to be important determinants of increased facility delivery [14]. It is important to note that the Cambodia Demographic and Health Surveys of 2000 [39] and 2005 [13] had already showed an increasing trend of facility deliveries in the country.

Two years of implementation is rather short to reveal the full effect of the voucher scheme. This innovative scheme usually needs time to refine its operation and overcome its shortcomings as highlighted by the operational analysis. The exclusion of twelve health centres that did not meet the selection criteria from the intervention automatically ruled out about 29% of poor pregnant women from the scheme. The failure of VMA staff to visit all villages as scheduled (to identify poor pregnant women and distribute vouchers) also deprived many poor pregnant women of the chance to receive vouchers. Moreover, many poor pregnant women who received vouchers did not use them for recommended maternal services at contracted facilities.

To improve the voucher scheme further, more efforts are needed to address the above-mentioned shortcomings. Instead of limiting vouchers to public health centres, the implementers could consider contracting qualified private providers for service delivery, at least in the catchment areas of the twelve excluded health centres. This would allow the voucher recipients to select a provider convenient to them, which might in turn increase their satisfaction and would also create competition among participating providers to improve the quality of their services [40]. However, this approach has no great potential in the study area, where there are very few qualified private providers who may moreover be reluctant to enter in a mutually acceptable contractual relationship. In addition, the Ministry of Health is not in favour of contracting private providers. The problem of voucher distribution could be solved by introducing pre-identification and further improving VMA performance and strengthening the role of village health volunteers in voucher distribution. In order to improve the utilisation of vouchers, the remaining barriers identified during the focus group discussions and key informant interviews should be addressed. The barriers related to health providers can be addressed through reinforcement of the contract and monitoring. To facilitate night transportation for pregnant women, a local arrangement should be developed by the communities. The project could also make use of local health volunteers by providing them with resources and incentives to arrange transport and company for pregnant women to deliver at health centres. Last but not least, more promotion of safe delivery at the public health facilities may further improve voucher utilisation.

## Conclusions

Despite some weaknesses in the methods, this study provides useful lessons for further improvement and scaling-up of vouchers and HEFs in Cambodia. The available evidence suggests that the combination of vouchers and HEFs, if carefully designed and implemented, has a strong potential for reducing financial barriers and hence improving access to skilled birth attendants for poor women. Nevertheless, these demand-side financing schemes have their own limitations. In the Cambodian context, vouchers and HEFs require other interventions, such as PBC and delivery incentive scheme, to improve provider performance to a level necessary for ensuring the supply of reasonable quality maternity services for potential users. Moreover, vouchers and HEFs cannot overcome many other non-financial barriers, such as distance, intra-household constraints and socio-cultural practices. To achieve the full potential of vouchers and HEFs, more efforts are needed to address their limitations, including the operational shortcomings of the voucher scheme in terms of distribution and utilisation. Voucher and HEF schemes can be scaled up to areas with reasonably good public health services, but close monitoring and evaluation are needed to ensure further improvement.

## Competing interests

The authors declare that they have no competing interests.

## Authors' contributions

PI processed and analysed the data and wrote the draft and final version of the manuscript. DH and NS collected data and contributed to the revised version of the manuscript. WVD provided content expertise throughout the design and implementation of the study and contributed to the final version of the manuscript. All authors read and approved the final manuscript.

## Pre-publication history

The pre-publication history for this paper can be accessed here:

http://www.biomedcentral.com/1471-2393/10/1/prepub

## Supplementary Material

Additional file 1 **Health Equity Fund: definition, questionnaire and eligibility criteria**. The file includes definition of a Health Equity Fund scheme, questionnaire and eligibility criteria used for interviews of potentially poor patients at hospitals to determine their eligibility for Health Equity Fund assistance. Click here for file

## References

[B1] United Nations United Nations Millenium Declaration, Resolution A/RES/55/2. New York 2000

[B2] Hill K Thomas K AbouZahr C Walker N Say L Inoue M Estimates of maternal mortality worldwide between 1990 and 2005: an assessment of available data Lancet 2007 370 1311 1319 10.1016/S0140-6736(07)61572-4 17933645

[B3] Ronsmans C Graham WJ Maternal mortality: who, when, where, and why Lancet 2006 368 1189 1200 10.1016/S0140-6736(06)69380-X 17011946

[B4] Hatt L Stanton C Makowiecka K Adisasmita A Achadi E Ronsmans C Did the strategy of skilled attendance at birth reach the poor in Indonesia? Bull World Health Organ 2007 85 774 782 10.2471/BLT.06.033472 18038059 PMC2636493

[B5] Donnay F Maternal survival in developing countries: what has been done, what can be achieved in the next decade Int J Gynaecol Obstet 2000 70 89 97 10.1016/S0020-7292(00)00236-8 10884537

[B6] Liljestr J Strategies to reduce maternal mortality worldwide Curr Opin Obstet Gynecol 2000 12 513 517 10.1097/00001703-200012000-00010 11128415

[B7] Campbell OM Graham WJ Strategies for reducing maternal mortality: getting on with what works Lancet 2006 368 1284 1299 10.1016/S0140-6736(06)69381-1 17027735

[B8] Chatterjee P Cambodia tackles high maternal mortality Lancet 2005 366 281 282 10.1016/S0140-6736(05)66967-X 16044532

[B9] Graham W Bell JS Bullough C Can skilled attendance at delivery reduce maternal mortality in developing countries? Lancet 2001 Antwerp, Belgium: ITG Press 97 129

[B10] Houweling TA Ronsmans C Campbell OM Kunst AE Huge poor-rich inequalities in maternity care: an international comparative study of maternity and child care in developing countries Bull World Health Organ 2007 85 745 754 18038055 10.2471/BLT.06.038588 PMC2636501

[B11] Sharma S Smith S Sonneveldt E Pine M Dayarantna V Sanders R Formal and informal fees for maternal health care services in five countries: policies, practices, and perspectives 2005 POLICY Project Washington DC: POLICY Working Paper Series No. 16

[B12] UNFPA Maternal and neonatal health in East and South-East Asia. Bangkok, Thailand 2006

[B13] Cambodia Demographic and Health Survey 2005 2006 Phnom Penh, Cambodia

[B14] UNFPA Cambodia Obstacles to Deliveries by Trained Health Providers to Cambodian Rural Women. PowerPoint Presentation http://www.un.org.kh/unfpa/docs/Obstacles.pdf accessed on 14-07-2009

[B15] Bhatia MR Gorter AC Improving access to reproductive and child health services in developing countries: are competitive voucher schemes an option? J Int Dev 2007 19 975 981 10.1002/jid.1361

[B16] Ensor T Consumer-led demand side financing in health and education and its relevance for low and middle income countries Int J Health Plann Manage 2004 19 267 285 10.1002/hpm.762 15387092

[B17] Gorter A McKay J Meuwissen L Segura Z Medina J Bellows B Targeting Vouchers to Underserved Populations in Nicaragua 2009 Washington, DC: Oral presentation in panel "Vouchers for Health", The Global Health Council's 36th Anual International Conference on Global Health

[B18] Prata N Graff M Graves A Potts M Avoidable maternal deaths: Three ways to help now Glob Public Health 2009 4 575 587 10.1080/17441690802184894 19326279

[B19] PSP-One Primer for Policymakers Vouchers for Health: A Focus on Reproductive Health and Family Planning Services 2006 PSP-One

[B20] Borghi J Ensor T Somanathan A Lissner C Mills A Mobilising financial resources for maternal health Lancet 2006 368 1457 1465 10.1016/S0140-6736(06)69383-5 17055948

[B21] Kundu F Claus P Janisch P Albrecht M Output-based Aid Vouchers: Increasing Facility Births for Poor in Kenya Proceedings of the Global Health Council's 36th Annual International Conference on Global Health: 26-30 May 2009, Washington, DC 2009 Oral presentation in panel "Vouchers for Health"

[B22] Onwujekwe O Hanson K Fox-Rushby J Inequalities in purchase of mosquito nets and willingness to pay for insecticide-treated nets in Nigeria: challenges for malaria control interventions Malar J 2004 3 6 10.1186/1475-2875-3-6 15023234 PMC395839

[B23] Rob U Rahman M Hena SA Increasing Access to Maternal Care through Vouchers in Bangladesh Proceedings of the Global Health Council's 36th Annual International Conference on Global Health: 26-30 May 2009, Washington, DC Oral presentation in panel "Vouchers for Health"

[B24] Worrall E Hill J Webster J Mortimer J Experience of targeting subsidies on insecticide-treated nets: what do we know and what are the knowledge gaps? Trop Med Int Health 2005 10 19 31 10.1111/j.1365-3156.2004.01355.x 15655010

[B25] Hardeman W Van Damme W Van Pelt M Por I Kimvan H Meessen B Access to health care for all? User fees plus a Health Equity Fund in Sotnikum, Cambodia Health Policy Plan 2004 19 22 32 10.1093/heapol/czh003 14679282

[B26] Jacobs B Price N Improving access for the poorest to public sector health services: insights from Kirivong Operational Health District in Cambodia Health Policy Plan 2006 21 27 39 10.1093/heapol/czj001 16293700

[B27] Noirhomme M Meessen B Griffiths F Ir P Jacobs B Thor R Improving access to hospital care for the poor: comparative analysis of four health equity funds in Cambodia Health Policy Plan 2007 22 246 262 10.1093/heapol/czm015 17526640

[B28] Meessen B Van Damme W Ir P Van Leemput L Hardeman W The New Deal in Cambodia: The second year. Confirmed results; confirmed challenges 2002 Phnom Penh, Cambodia: MSF Cambodia

[B29] Van Damme W Meessen B von Schreeb J Thayly H Thomé JM Overtoom R Sotnikum New Deal: The first year. Better income for health staff; better service to the population 2001 Phnon Penh, Cambodia: MSF Cambodia

[B30] Bloom E King E Bhushan I Kremer M Clingingsmith D Loevinsohn B Contracting for Health: Evidence from Cambodia. An evaluation report http://www.brookings.edu/views/papers/kremer/20060720cambodia.pdf accessed on 05-10-2009

[B31] Soeters R Griffiths F Improving government health services through contract management: a case from Cambodia Health Policy Plan 2003 18 74 83 10.1093/heapol/18.1.74 12582110

[B32] Ensor T Cooper S Overcoming barriers to health service access: influencing the demand side Health Policy Plan 2004 19 69 79 10.1093/heapol/czh009 14982885

[B33] O'Donnell O Access to health care in developing countries: breaking down demand side barriers Cad Saude Publica 2007 23 2820 2834 10.1590/S0102-311X2007001200003 18157324

[B34] Palmer N Mueller DH Gilson L Mills A Haines A Health financing to promote access in low income settings-how much do we know? Lancet 2004 364 1365 1370 10.1016/S0140-6736(04)17195-X 15474141

[B35] Bigdeli M Annear PL Barriers to access and the purchasing function of health equity funds: lessons from Cambodia Bull World Health Organ 2009 87 560 564 10.2471/BLT.08.053058 19649372 PMC2704035

[B36] Wilkinson D Holloway J Fallavier P The Impact of User Fees on Access, Equity and Health Provider Practices in Cambodia 2001 Phnom Penh, Cambodia: Ministry of Health

[B37] Barber S Bonnet F Bekedam H Formalizing under-the-table payments to control out-of-pocket hospital expenditures in Cambodia Health Policy Plan 2004 19 199 208 10.1093/heapol/czh025 15208276

[B38] Making pregnancy safer: the critical role of the skilled attendant. A joint statement by WHO, ICM and FIGO 2004 Geneva: Department of Reproductive Health and Research, World Health Organization

[B39] Cambodia Demographic and Health Survey 2000 2001 Phnom Penh, Cambodia

[B40] Sandiford P Gorter A Rojas Z Salvetto M A guide to competitive vouchers in health 2005 Washington DC: Private Sector Advisory Unit, the World Bank Group

