# Supplementary material for: Using targeted vouchers and health equity funds to improve access to skilled birth attendants for poor women: a case study in three rural health districts in Cambodia
Source: BMC Pregnancy Childbirth. 2010 Jan 7;10:1. doi: 10.1186/1471-2393-10-1 (PMC2820432; doi:10.1186/1471-2393-10-1)
Supplement: Additional file 1 — Health Equity Fund: definition, questionnaire and eligibility criteria. The file includes definition of a Health Equity Fund scheme, questionnaire and eligibility criteria used for interviews of potentially poor patients at hospitals to determine their eligibility for Health Equity Fund assistance. [file 1471-2393-10-1-S1.PDF]

Additional file 1

Definition of a Health Equity Fund scheme

HEF is a demand-side financing mechanism to promote access to priority public health services for the poor in an environment where user fees are charged. The management of the fund is entrusted to a third party, usually a local non-governmental organisation. HEF beneficiaries are identified according to eligibility criteria, either at the community before health care demand (pre-identification) or at the health facilities through interviews (post-identification). At the health facility, the eligible poor patients get full or partial support from HEF for the cost of user fees, transport cost and other costs during hospitalisation.

Questionnaire and eligibility criteria for Health Equity Fund

**Q1 – Housing**	**Score**	**Q6 – Farm assets**	**Score**
A. Roof: Thatched/Leave/Tent	0	A. None	0
Tiled/Zinc/Tin sheet	1	B. Plow	1
B. Wall: None/Leave/Bamboo	0	C. Draft animals (oxen, baffeloes)	2
Wood	1	D. Water pump	3
Cement	2	F. Tractor/ Tiller machine	4
C. Floor: None	0	**Q7 – Livestock**	**Score**
Bamboo	1	A. None	0
Wood	2	B. 1 adult pig/<30Chickens/Ducks	1
Cement/Tile	3	C. 2 Adult pigs/>30Chickens/Ducks	2
D. Condition: Bad	0	D. >2Goats/1 cow/ox/buffalo	3
Good	1	E. >2 Oxen/ buffaloes/horses	4
Very good	2		
**Q2 – Electronic tools**	**Score**	**Q8 – Cash income/Person/Day)**	**Score**
A. None , Radio	0	A. <2,000 Riels*	0
B. Tape/TV (Black & White)	1	B. 2,000R to 4,000 Riels*	1
C. TV (Color)	2	C. 4,100R to 8,000 Riels*	2
D. ICOM Radio/Cell phone	3	D. 8,100R to 16,000 Riels*	3
		E. >16,000 Riels*	4
**Q3 – Electricity**	**Score**	**Q9 – Dependents**	**Score**
A. None, Kerosene	0	A. >2 Elderly/Disable/Orphans	0
B. Battery < 50 Ampere	1	B. One Elderly/Disable/Aphelion	1
C. Electric buying	2	C. None	2
D. Owner ship of generator	3		
**Q4 – Transportation means**	**Score**	**Q10 – Length of severe illness last year**	**Score**
A. None	0	A. >30 days	0
B. Bike/ Small Boat	1	B. 15-30 days	1
C. Horse/Oxcart	2	C. 5-15 days	2
D. Motor boat/Motorbike	3	D. <5 days	3
E-Vehicle/Power Tiller	4		
**Q5 – Productive Lands**	**Score**	**Q11 – Household health expenditure last year**	**Score**
**Size** A-None	0	A. >500,000 Riels*	0
B-< 01 Hectare	1	B. 200,000 to 500,000 Riels*	1
C- 01-02 Hectares	2	C. <200,000 Riels*	2
D- >02 to 05 Hectares	3		
E- > 05 Hectares	4		
**Quality** A-Third Category	0	**Q12 – Borrowing for health care**	**Score**
B- Second Category	1	A. Used to borrow for health care	0
C- First Category	2	B. Never borrow	1

TOTAL SCORE AND ELIGIBILITY CRITERIA

A:	Score between	0-10	Very poor
B:	Score between	11-14	Poor
C	Score equal or above	15	Non-poor and rejected from support

* ***Riel is Cambodian currency. The exchange rate is around 4,000 Riels = USD1***
